# Frequency of adult type-associated lactase persistence LCT-13910C/T genotypes in the Czech/Slav and Czech Roma/Gypsy populations

**DOI:** 10.1590/1678-4685-GMB-2016-0071

**Published:** 2017-05-11

**Authors:** Jaroslav A. Hubácek, Věra Adámková, Lenka Šedová, Věra Olišarová, Václav Adámek, Valérie Tóthová

**Affiliations:** 1 Centre for Experimental Medicine, Institute for Clinical and Experimental Medicine, Prague, Czech Republic; 2 Department of Preventive Cardiology, Institute for Clinical and Experimental Medicine, Prague, Czech Republic; 3 University of South Bohemia in České Budějovice, Faculty of Health and Social Studies, České Budějovice, Czech Republic; 4 Czech Technical University of Prague, Faculty of Biotechnical Enginnering, Kladno, Czech Republic

**Keywords:** Lactose, lactase persistence, polymorphism, Czech/Slav, Czech Gypsy/Roma

## Abstract

Lactase non-persistence (leading to primary lactose intolerance) is a genetically dependent inability to digest lactose in adulthood. As part of the human adaptation to dairying, the human lactase *LCT*-13910C/T mutation (which propagates adult expression of lactase) developed, spread and participated in the adaptation to dairying. This variant is associated with lactase activity persistence, and its carriers are able to digest lactose. We compared the frequencies of lactase 13910C/T (rs4988235) genotypes in Czechs/Slavs (N = 288) and Czech Gypsies/Roma (N = 300), two ethnically different groups where this polymorphism has not yet been analysed. Allelic frequencies significantly differed between the populations (p < 0.0001). In Czechs/Slavs, the lactase persistence T allele was present in 76% of the individuals, which is in agreement with frequencies among geographically neighbouring populations. In the Czech Gypsy/Roma population, only 27% of the adults were carriers of at least one lactase persistence allele, similar to the Indian population. In agreement with this result, dairy product consumption was reported by 70.5% of Czechs/Slavs and 39.0% of the Czech Gypsy/Roma population. Both in the Czech Gypsy/Roma and in the Czech/Slavs populations, the presence of carriers of the lactase persistence allele was similar in subjects self-reporting the consumption of unfermented/fresh milk, in comparison to the others.

Lactase (lactase-phlorizin hydrolase–enzyme activities as described in EC 3.2.1.23 and EC 3.2.1.62) is an enzyme that cleaves disaccharide lactose (obtained in milk at about 4%) into two simple sugars, glucose and galactose, both of which, unlike lactose, can be absorbed. Lactase non-persistence (OMIM acc No. 223100) is a time-dependent down-regulation of lactase enzyme activity in the small intestine. Lactase non-persistence leads to primary lactose intolerance and usually occurs between early childhood and puberty. It leads to physiological problems like flatulence, bloating and diarrhoea (Flatz and Rotthauwe, 1977).

Lactase is coded by the *LCT* gene (OMIM acc No. 603202). Lactase persistence is associated with the variant rs4988235 (-13910C/T), which is located within the upstream neighbouring gene, *MCM6* (minichromosome maintenance complex component 6, OMIM acc No. 601806). The region within the *MCM6* gene acts as a *cis*-element, which enhances differential transcriptional activation of the lactase promoter (Olds and Sibley, 2003). Altogether, there are five different point mutations within the *MCM6* region, which seems to be responsible for the regulation of *LCT* gene expression (Gerbault *et al.*, 2013).

Lactase persistence mutations are not evenly distributed across ethnic groups (Mattar *et al.*, 2012; Gerbault *et al.*, 2013), but they appear to reflect the ancient history of dairying among populations and of migration events. In Europeans, the C → T exchange at position −13,910 on the *LCT* gene (rs4988235) is the most common (Mattar *et al.*, 2012) lactase persistence variant and has been confirmed to be functional *in vivo* (Fang *et al.*, 2012).

Many (but not only) European individuals are carriers of the mutated allele and retain the ability to digest lactose through their entire lifespans. The reason for the advantageousness of this mutation is still debatable, but it is believed that it is an adaptation to settlements, the domestication of cattle, milk consumption (not only as a continuous source of energy, but also as an important non-seasonal source of calcium and vitamin D), and consumption of other dairy products led to the wide and rapid expansion of this mutation (Gerbault, 2013; Gerbault *et al.*, 2013; Lachance and Tishkoff, 2013).

In our study, we analysed the prevalence of *LCT* rs4988235 genotypes in Czechs/Slavs (non-Roma) and Czech Gypsies/Roma, given that within these ethnic groups no information has so far been reported on the distribution of the lactase persistence allele.

Six-hundred-and-one unrelated adults (at least 18 years old at the time of examination) were included in the study. Czech Gypsies/Roma (N = 302) were recruited using snowball sampling (Hughes *et al.*, 1995) and 298 Czechs/Slavs using quota sampling (for more details, see Adámková *et al.*, 2015; Šedová *et al.*, 2015; Tóthová *et al.*, 2015), where gender (50:50%) was used as a quota. Ethnicity was based on self-reported information. All subjects completed a one-day dietary record and the percentage of cases of milk and/or dairy product consumption/non-consumption was calculated. Written informed consent was provided by all subjects enrolled in the study. The study was approved by the institution's ethics committee and conducted according to Good Clinical Practice guidelines.

DNA was isolated from DNA buccal swabs using the XTREME DNA kit (both Isohelix, Cell Project Ltd, UK) according to the manufacturer's instructions.

Rs4988235 genotypes were analysed using PCR-RFLP (Mulcare *et al.*, 2004). Briefly, oligonucleotides 5'-gct ggc aat aca gat aag ata atg ga-3 and 5'-ctg ctt tgg ttg aag cga aga t-3 were used for DNA amplification. All PCR chemicals were obtained from Fermentas International, Inc. (Burlington, ON, Canada) and PCR reactions were performed on an MJ Research DYAD Disciple PCR device. The PCR product (201 bp) was digested by 5U of the restriction enzyme *Hinf*I (Fermentas International Inc., Burlington, ON, Canada) overnight at 37 °C. Restriction fragments were separated using 10% polyacrylamide gels based on the MADGE system (Day *et al.*, 1996). PCR products were cut out as 177 bp and 24 bp restriction fragments where the lactose persistence allele T was present.

Deviations from the Hardy-Weinberg equilibrium were analysed using an online calculator (Court 2005-2008) and the differences in allele and genotype frequencies were compared using a chi-square test (Kirkman, 1996) in dominant, codominant and recessive models.

We successfully genotyped 288 (96.0%) Czechs/Slavs (147 males and 141 females) and 300 (99.3%) Czech Gypsies/Roma (151 males and 149 females) for the rs4988235 variant. In both groups, genotype distributions were within the Hardy-Weinberg equilibrium (p = 0.81 for Czechs/Slavs and p = 0.94 for Czech Gypsies/Roma). We observed no gender differences within ethnic groups in allele/genotype frequencies.

The prevalence of rs4988235 genotypes was significantly different (p < 0.0001 for the codominant model of analysis) between the two ethnic groups under examination (Table 1). Significant differences observed also for dominant p < 0.0001) and recessive (p < 0.0001) models of analysis.

**Table 1:** Frequency of LCT −13910C/T (rs4988235) genotypes in the Czech/Slav and Czech Gypsy/Roma populations.

	Caucasians		Gypsies/Roma	
Rs4988235	N	%	N	%
CC	68	23.6	220	73.3
CT	146	50.7	74	24.7
TT	74	25.7	6	2.0
P < 0.0001				
C	282	49.0	514	85.7
T	294	51.0	86	14.3
P < 0.0001				

Within the Czech/Slav population, 23.6% of the individuals had the lactase CC non-persistence genotype. In contrast, in Czech Gypsy/Roma individuals, carriers of the lactase non-persistence CC genotype accounted for the vast majority of the population, specifically 73.3% of individuals. This finding is in agreement with the prevalence for milk/milk products consumption. All dairy consumption among Czechs/Slavs was reported at 70.5% but at only 37.3% among Czech Gypsies/Roma (p < 0.0005).

Finally no differences between ethnic groups were observed, also in cases where unfermented/fresh milk consumers *vs.* others were analysed. No differences in genotype frequencies were detected in Czechs/Slavs (p = 0.43) or Czech Gypsies/Roma (p = 0.14). In Czechs/Slavs, the frequency of the lactase persistence allele is almost identical to two geographically close populations, namely Poles (Kuokkanen *et al.*, 2005; Madry *et al.*, 2010) and Austrians (Tag *et al.*, 2008).

It is noteworthy that by the use of computer simulation (Itan *et al.*, 2009), the origin of the *LCT* gene mutation at position −13910 is pinpointed in Central Europe roughly 7,000-8,000 years ago. This points to a huge (although probably still not fully understood) advantage of this mutation, since, especially in Northern Europe, around 80% of the population are carriers of at least one mutated allele.

In contrast, the frequency of this allele in Czech Gypsies/Roma is very similar to two Indian populations (Mattar *et al.*, 2012). As mentioned elsewhere, the ancestry of European Roma/Gypsies is traceable to India (Mendizabal *et al.*, 2011), and our results further confirm this geographical origin. Furthermore, our study highlights the non-assimilation of this ethnic group, which is in contrast with the observations of Hungarians, another central European population of Asian origin (Nagy *et al.*, 2011). A study of medieval samples (Krüttli *et al*., 2014) of German origin suggests that genetically dependent lactase persistence in Central Europe occurred as early as circa 1200 AD.

This is the first report to focus on the presence of the lactase persistence allele of the rs4988235 (C-13910T, *LCT* gene) polymorphism within the Czech/Slav and Czech Roma/Gypsy populations. The differences we observed point to the origin of both ethnic groups and to the non-assimilation of Roma/Gypsies within the European region. Finally, in both groups, carriers of the lactase persistence allele occur similarly among consumers of unfermented milk and others.

## References

[B1] Adámkova V, Hubacek JA, Nováková D, Dolák F, Adámek V, Lánská V, Tóthová V, Šedová L (2015). Genetic and biochemical characteristics in the Roma minority in the
South Bohemia Region. Neuro Endocrinol Lett.

[B2] Day IN, Bolla M, Haddad L, O'dell S, Humphries SE (1996). Microtiter Array Diagonal Gel Electrophoresis (MADGE) for population
scale genotype analyses. Methods Mol Med.

[B3] Fang L, Ahn JK, Wodziak D, Sibley E (2012). The human lactase persistence-associated SNP −13910*T enables in vivo
functional persistence of lactase promoter reported transgene
expression. Hum Genet.

[B4] Flatz G, Rotthauwe HW (1977). The human lactase polymorphism: Physiology and genetics of lactose
absorption and malabsorption. Prog Med Genet.

[B5] Gerbault P (2013). The onset of lactase persistence in Europe. Hum Hered.

[B6] Gerbault P, Roffet-Salque M, Evershed RP, Thomas MG (2013). How long have adult humans been consuming milk?. IUBMB Life.

[B7] Hughes AO, Fenton S, Hine CE, Pilgrim S, Tibbs N (1995). Strategies for sampling black and ethnic minority
populations. J Publ Health Med.

[B8] Itan Y, Powel A, Beaumont MA, Burger J, Thomas MG (2009). The origins of lactase persistence in Europe. PLoS Comput Biol.

[B9] Krüttli A, Bouwman A, Akgül G, Della Casa P, Rühli F, Warinner C (2014). Ancient DNA analysis reveals high frequency of European lactase
persistence allele (T-13910) in medieval central europe. PLoS One.

[B10] Kuokkanen M, Butzow R, Rasinperä H, Medrek K, Nilbert M, Malander S, Lubinski J, Järvelä I (2005). Lactase persistence and ovarian carcinoma risk in Finland, Poland and
Sweden. Int J Cancer.

[B11] Lachance J, Tishkoff SA (2013). Population genomics of human adaptation. Annu Rev Ecol Evol Syst.

[B12] Madry E, Lisowska A, Kwiecien J, Marciniak R, Korzon-Burakowska A, Drzymala-Czyz S, Mojs E, Walkowiak J (2010). Adult-type hypolactasia and lactose malabsorption in
Poland. Acta Biochim Pol.

[B13] Mattar R, de Campos Mazo DF, Carrilho FJ (2012). Lactose intolerance: Diagnosis, genetic, and clinical
factors. Clin Exp Gastroenterol.

[B14] Mendizabal I, Valente C, Gusmão A, Alves C, Gomes V, Goios A, Parson W, Calafell F, Alvarez L, Amorim A (2011). Reconstructing the Indian origin and dispersal of the European Roma: A
maternal genetic perspective. PLoS One.

[B15] Mulcare CA, Weale ME, Jones AL, Connell B, Zeitlyn D, Tarekegn A, Swallow DM, Bradman N, Thomas MG (2004). The allele of a single-nucleotide polymorphism 13.9 kb upstream of the
lactase gene (LCT) (C-13.9 kbT) does not predict or cause the lactase-persistence
phenotype in Africans. Am J Hum Genet.

[B16] Nagy D, Tömöry G, Csányi B, Bogácsi-Szabó E, Czibula Á, Priskin K, Bede O, Bartosiewicz L, Downes CS, Raskó I (2011). Comparison of lactase persistence polymorphism in ancient and
present-day Hungarian populations. Am J Phys Anthropol.

[B17] Olds LC, Sibley E (2003). Lactase persistence DNA variant enhances lactase promoter activity in
vitro: Functional role as a cis regulatory element. Hum Mol Genet.

[B18] Šedová L, Tóthová V, Olišarová V, Adámkova V, Bártlová S, Dolák F, Kajanová A, Mauritzová I, Nováková D, Prokešová R (2015). Evaluation of selected indicators of overweight and obesity of Roma
minority in the region of South Bohemia. Neuro Endocrinol Lett.

[B19] Tag CG, Oberkanins C, Kriegshäuser G, Ingram CJ, Swallow DM, Gressner AM, Ledochowski M, Weiskirchen R (2008). Evaluation of a novel reverse-hybridization StripAssay for typing DNA
variants useful in diagnosis of adult-type hypolactasia. Clin Chim Acta.

[B20] Tóthová V, Bártlová S, Šedová L, Olišarová V, Prokešová R, Adámkova V, Mauritzová I, Treslova M, Chloubová I, Mikšová Z (2015). The importance of self-management in the prevention and treatment of
excessive weight and obesity. Neuro Endocrinol Lett.

